# Establishing an ICD-10 code based SARI-surveillance in Germany – description of the system and first results from five recent influenza seasons

**DOI:** 10.1186/s12889-017-4515-1

**Published:** 2017-06-30

**Authors:** S. Buda, K. Tolksdorf, E. Schuler, R. Kuhlen, W. Haas

**Affiliations:** 1 Robert Koch Institute, Department for infectious disease epidemiology, Respiratory infections unit, Seestr. 10, 13353 Berlin, Germany; 2 HELIOS KLINIKEN GmbH, Friedrichstraße 136, 10117 Berlin, Germany

**Keywords:** Influenza, Hospital surveillance, Severe acute respiratory infections, ICD-10-codes

## Abstract

**Background:**

Syndromic surveillance of severe acute respiratory infections (SARI) is important to assess seriousness of disease as recommended by WHO for influenza. In 2015 the Robert Koch Institute (RKI) started to collaborate with a private hospital network to develop a SARI surveillance system using case-based data on ICD-10 codes. This first-time description of the system shows its application to the analysis of five influenza seasons.

**Methods:**

Since week 40/2015, weekly updated anonymized data on discharged patients overall and on patients with respiratory illness including ICD-10 codes of primary and secondary diagnoses are transferred from the network data center to RKI. Retrospective datasets were also provided. Our descriptive analysis is based on data of 47 sentinel hospitals collected between weeks 1/2012 to 20/2016. We applied three different SARI case definitions (CD) based on ICD-10 codes for discharge diagnoses of respiratory tract infections (J09 - J22): basic CD (BCD), using only primary diagnoses; sensitive CD (SCD), using primary and secondary diagnoses; timely CD (TCD), using only primary diagnoses of patients hospitalized up to one week. We compared the CD with regard to severity, age distribution and timeliness and with results from the national primary care sentinel system.

**Results:**

The 47 sentinel hospitals covered 3.6% of patients discharged from all German hospitals in 2013. The SCD comprised 2.2 times patients as the BCD, and 3.6 times as many as the TCD. Time course of SARI cases corresponded well to results from primary care surveillance and influenza virus circulation. The patients fulfilling the TCD had been completely reported after 3 weeks, which was fastest among the CD. The proportion of SARI cases among patients was highest in the youngest age group of below 5-year-olds. However, the age group 60 years and above contributed most SARI cases. This was irrespective of the CD used.

**Conclusions:**

In general, available data and the implemented reporting system are appropriate to provide timely and reliable information on SARI in inpatients in Germany. Our ICD-10-based approach proved to be useful for fulfilling requirements for SARI surveillance. The exploratory approach gave valuable insights in data structure and emphasized the advantages of different CD.

## Background

Syndromic influenza surveillance in Germany is based on a network of primary care physicians that report consultations for acute respiratory infections (ARI) [1]. Weekly reports of ARI activity and corresponding results from virological sentinel surveillance are published year-round and data are sent to ECDC and WHO for inclusion in the international influenza surveillance networks (EISN, FluID, FluNet [2, 3]). This sentinel system (Working Group Influenza/Arbeitsgemeinschaft Influenza (AGI)) has proven its value in providing timely and reliable information on influenza activity in Germany and the burden of disease, especially during the pandemic 2009 [4–6].

However, during the process of pandemic preparedness planning and especially since the influenza pandemic 2009 the need to implement a routine sentinel surveillance system for severe acute respiratory infections (SARI) became obvious [7, 8]. In the course of the pandemic three ad hoc systems were established to capture severe respiratory cases. The first was the Pandemic Hospital Based Surveillance (Pandemische Influenza Krankenhaus-Surveillance (PIKS)) that was implemented by the Robert Koch Institute (RKI) to collect data on hospital admissions diagnosed with laboratory confirmed influenza [9]. The second was a SARI-Surveillance study that was part of a hospital-based pandemic influenza vaccine effectiveness study [10]. Lastly, there was enhanced surveillance of influenza during the pandemic with systematic collection of additional variables supplementary to the national regular mandatory notifications of laboratory confirmed influenza cases. For the mandatory system, it was only possible to record information on severity and risk factors according the WHO recommendations as long as the pandemic was under the scope of the Public Health Emergency of international Concern (PHEIC [11]) and an additional national pandemic notification ordinance was in force [12]. Although the information gathered on severe influenza cases and risk factors for severe course of disease were not only important in the national context but also used for global analyses [13], none of the three approaches were continued on a routine basis. Thus, the aim to establish a sustainable, timely and cost-efficient approach that could collect data during seasonal influenza epidemics and pandemics remained a priority.

The WHO recommends the national development of SARI surveillance for hospital inpatients for influenza surveillance. The approach combines a syndromic surveillance part, where severe acute respiratory illness is monitored, and a virological surveillance part, where all or a systematically selected subset of patients were tested for influenza [14]. While European countries have a long-standing tradition of national outpatient syndromic influenza surveillance systems, only a few have established hospital inpatient SARI surveillance systems [15]. Other European countries concentrate on laboratory-confirmed influenza cases admitted to hospitals or intensive care units only [2, 16].

The maintenance of continuous influenza surveillance systems requires sufficient financial and personnel resources. Therefore approaches using secondary data offer an attractive possibility [17]. In 2015, the RKI set up a research collaboration with a private network of hospitals in order to develop a SARI sentinel surveillance system. This used case-based data coded according to the International Statistical Classification of Diseases and Related Health Problems 10th Revision (ICD-10) [18] and additional information on relevant procedures such as ventilation. A similar approach was used previously in the German primary care syndromic sentinel system to integrate case based ICD-10 coded reports. This led to fundamental improvements as it provided more detailed information on single ARI cases such as age, sex and single respiratory diagnosis [19, 20].

We described the establishment of an ICD-10-based inpatient syndromic sentinel system and its application to the analysis of five influenza seasons. We compared the impact of different case definitions on the ability to capture SARI cases, to allow a timely trend analysis of the seasonal epidemic and to reflect the burden caused by influenza when compared to routine outpatient surveillance.

## Methods

### Description of the system

The syndromic SARI surveillance is based on anonymized patient data originating from quality assurance reports from hospitals belonging to the HELIOS Kliniken GmbH. The data use and reporting procedure was approved by both the RKI and HELIOS Kliniken GmbH data protection authority. As the study involved no interventions and the analysis at RKI was based on anonymized data only, no ethical clearance was required in accordance with section 15 paragraph 1 of the Professional Code for Physicians in Berlin, in accordance with section 15 paragraph 1 of the Model Professional Code for Physicians in Germany [21]. Therefore, data use has not been discussed with an ethical committee. A scientific cooperation agreement was put in place between the RKI and HELIOS Kliniken GmbH which set out the data that was to be shared. Retrospective datasets for the years 2009 to 2014 were provided to the RKI in August 2015. From season 2015/2016 (week 40/2015) onwards, cumulative datasets containing data on all patients discharged were transferred via secure web service from the HELIOS data center to RKI within two weekdays of a weekly cut-off date. Updated datasets were then available for analyses the following day. These annual datasets include data from 39 hospitals in 2009, 47 hospitals in 2012 and 84 hospitals in 2016. Historical and current data were stored on a SQL server with restricted access only to a few selected members of the Unit for Respiratory Infections at RKI.

The weekly updated data reports consist of two data sets. The first dataset describes the total number of hospitalizations within the reporting sentinel sites and contains admission and discharge date, age (in years), sex and the first two digits of the patient’s 5-digit post code (indicates region and is usually able to identify the individual German federal state) of every hospital inpatient. This dataset also contains information on duration of hospital stay, duration of stay in the intensive care unit (ICU) and mode of discharge (discharged home, discharged to other facility or deceased). The second dataset describes the subset of patients with any respiratory ICD-10 diagnosis code from chapter X (Diseases of the respiratory system J00 - J99) recorded in their main or secondary diagnosis. This second dataset also providesinformation on admission diagnosis, on the primary and all secondary discharge diagnoses, on duration of ventilation, the first three digits of the patient’s 5-digit post code, and the main department in hospital where the patient received their care. A MD5 hash code was used as an anonymous identifier in both datasets, which enabled an evaluation of repeated stays of patients in the same hospital but didn’t allow identification at RKI.

### Study population and selection of diagnosis codes

**Fig. 1:**
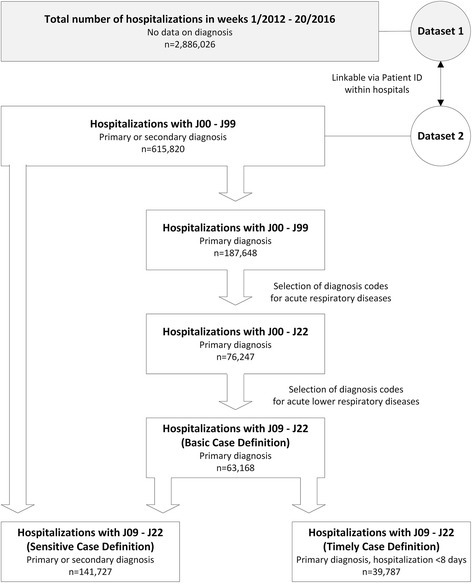
Flow chart on data selection and generation of case definitions (data from week 1/2012–20/2016)

Yearly datasets from 2012 onwards have been validated. For the analysis in this paper we only included data from the 47 hospital sites for which we had annual data for every year in the period of week 1/2012–20/2016 in order to illustrate the characteristics of our data over five consecutive influenza seasons. This was last updated on 27 June 2016. The number of patients covered by the sentinel sites were compared to nationwide data from the federal statistical office of Germany regarding the number of hospitals and the number of hospitalized patients in the years 2013 and 2014 [22, 23]. In the pilot phase several case definitions for SARI syndrome based on combinations of ICD-10 J-codes [18] in discharge diagnoses were selected after a literature research (Appendix, [24–28]) and tested with regard to their sensitivity for surveillance purposes (Fig. 1). Our dataset includes patients with any primary and secondary ICD-10 diagnosis code from J00 to J99. Thus, combinations of ICD-10 codes containing codes from other chapters are not feasible (Appendix, [25, 27]). As the focus in syndromic influenza surveillance is on acute respiratory diseases, we restricted probable cases to ICD-10 diagnosis codes from J00 to J22 and excluded ICD-10 codes from J40 to J47, as they are used for chronic lower respiratory diseases (Appendix, [24]). We described the occurrence of the ICD-10 diagnosis groups J00 - J06 (acute upper respiratory infections), J09 - J18 (influenza and pneumonia) and J20–22 (other acute lower respiratory infections) in the sentinel data and the age distributions within the diagnosis groups. We defined five age groups according to the outpatient sentinel system (AGI) [5, 6], which were 0–4 years, 5–14 years, 35–59 years and 60 years or older.

We subsequently chose to exclude the diagnosis group for acute upper respiratory infections J00 - J06, as it does not represent characteristics of severe disease and tends to be associated with hospitalizations mainly in very young children. In our data, 37% of the patients with primary diagnosis J00 - J06 were under 3 years old (data not shown). In the following text we defined SARI cases using ICD-10 diagnosis codes J09 – J22. This case definition aims to capture both the influenza-related pneumonias and other influenza-related acute infections of the lower respiratory tract [28]. An analogous selection of ICD-10 diagnosis codes has been described before [26].

As the diagnosis group J09 - J18 is the most relevant for influenza surveillance, we split the group into J09 - J11 (influenza) and J12 - J18 (pneumonia) and described the occurrence of those groups and their age distribution. We graphically compared the subgroup of influenza diagnoses (J09 - J11) with the much larger group of acute lower respiratory diseases (J09 - J22).

To compare characteristics of case definitions, the terms in-season and off-season were used. Weeks with influenza activity (in-season) were defined according to the criteria of the AGI (week 50/2012–16/2013, week 8/2014–14/2014, week 2/2015–16/2015, week 2/2016–15/2016), the remaining weeks were defined as off-season [5, 6]. In order to differentiate increased proportions of SARI cases supposedly due to intense transmission from higher rates during holidays (potential artifacts), we examined the weeks of Christmas Holidays seperately in our analysis. As the dates for the Christmas school holidays varied among federal states, we defined weeks including any of the national holidays such as the 24th to 26th December, the 31th December and the 1st of January as Christmas Holidays in this paper. Weekly numbers of cases were calculated using the admission date and the discharge diagnoses of patients.

### Case definitions

Three different case definitions were developed.

We included patients containing any ICD-10 code of the group J09 - J22 (SARI) in their primary discharge diagnosis in our *basic case definition (BCD)*.

In order to generate a highly sensitive estimate of the burden of severe acute respiratory disease, we also generated a *sensitive case definition (SCD)*. It comprised patients with any ICD-10 code of the group J09 - J22 in their primary or secondary discharge diagnoses.

A third case definition was used to maximize the timeliness of information on SARI trends during the season. As weekly data reports only include information on patients who have been discharged, we restricted analysis to patients with hospital stays of one week duration or less. Additionally, we only included patients with an ICD-10 code from the group J09 - J22 in the primary discharge diagnosis, as these patients tended to be discharged within a shorter time frame (*timely case definition, TCD*) (Fig. 1).

We illustrated time course and severity of recent influenza seasons and showed characteristics in the three different case definitions graphically. We calculated the weekly proportions of hospitalizations, ICU admissions and deaths associated with SARI separately for all three case definitions and displayed the time trend of proportions graphically. Proportions were calculated as described before [28].

In order to quantify the time lag in data with respect to the three case definitions, we looked at cases with reported admission date in week 6/2016. The reference dataset used was the week 16/2016 at which point we assumed that all patients would have been discharged.We then analyzed the datasets from weeks 7/2016, 8/2016, 9/2016 to calculate the percentage of reported cases with admission date from week 6/2016. This provided us with an estimate of completeness of case ascertainment at 2, 3 and 4 weeks after admission.

We compared the course of the TCD with medically attended acute respiratory infections in outpatients per 100,000 population per week (MAARI incidence) and corresponding virological data generated in the German primary care sentinel system (AGI) [6].

Maps were generated using RegioGraph Analyse 13; StataSE 14, Microsoft Visio and Microsoft Excel were used for other graphs and analyses.

## Results

### Distribution of sentinel hospitals and coverage of the German population in the years 2013 and 2014

**Fig. 2:**
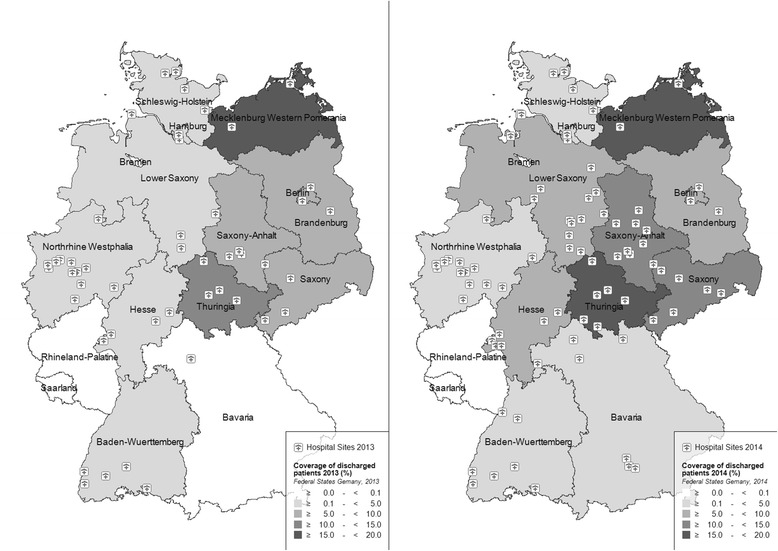
Regional distribution of sentinel sites in 2013 and in 2014

In 2013, the 47 sentinel sites included 2.4% of all hospitals in Germany and were located in 13 of the 16 federal states. There were no sentinel sites in the federal states of Bremen, Rhineland-Palatine and Saarland. In the year 2014, an additional 35 sites became part of the sentinel network, thus covering 4.2% of the German hospitals in 2014 (Fig. 2). The sentinel hospitals accounted for 3.6% of all patients discharged in any German hospital in 2013, and 5.9% in 2014 [18, 23]. However, only data from 47 hospitals contributing since 2012 were used for the description of the system.

### Study population and selection of ICD-10 discharge diagnosis groups for SARI

**Fig. 3:**
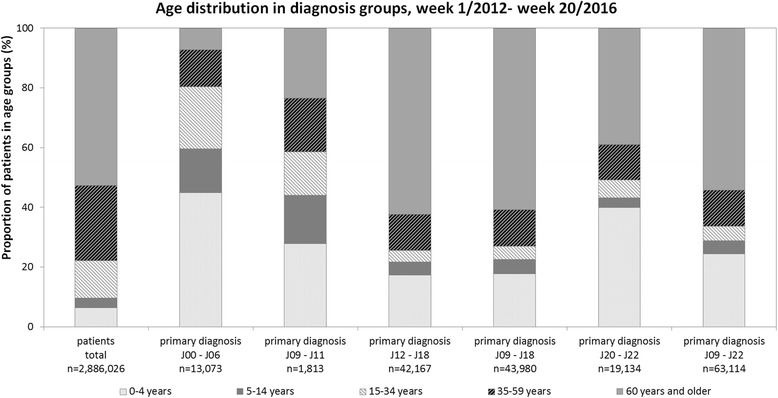
Age distribution of patients overall and for different ICD-10 diagnosis groups in weeks 1/2012–20/2016

Of the patients admitted to the 47 hospitals during the study period (week 1/2012–20/2016) 53% were 60 years or older. However, this age group only accounted for 7% of acute upper respiratory disease diagnoses (J00 - J06). In this diagnosis group children, especially young children below 5 years of age, comprised 45% of cases (Fig. 3).

The most relevant diagnosis groups for syndromic surveillance of influenza and pneumonia are covered by ICD-10 codes J09 – J11 (influenza) and J12 – J18 (pneumonia). The number of cases in the subgroup of influenza diagnoses (J09 - J11) was small compared to the number of cases in the group of pneumonia diagnoses (J12 – J18). Age was distributed more evenly for patients with a diagnosis of influenza (J09 - J11) compared to other diagnostic groupings. There were 28% and 23% in the youngest and the oldest age groups, respectively and 15% to 18% in all other age groups. The group of pneumonia diagnoses (J12 - J18) had a large proportion of elderly patients (60 years and older) with 61%. Young children up to 4 years of age accounted for 18% of pneumonia diagnosis group (J12 - J18). When patients with the primary discharge diagnoses of influenza and pneumonia (J09 – J18) were considered as a whole they had had the same age distribution as the pneumonia group (J12 – J18) due to the low numbers of the J09 – J11 group. In the diagnosis group of other acute lower respiratory infections (IDC-10 codes J20 - J22) the youngest and the oldest age groups contributed 40% and 39%, respectively. In the study period, 54% of the patients with ICD-10 codes J09 - J22 (SARI) in their primary diagnosis were at least 60 years old, 25% were less than 5 years old, and 12% were between 35 and 59 years old. This is the grouping that was applied for the basic case definition. The use of primary diagnoses codes enables comparisons with health statistics from other sources such as the Federal Statistical Office in Germany.

**Fig. 4:**
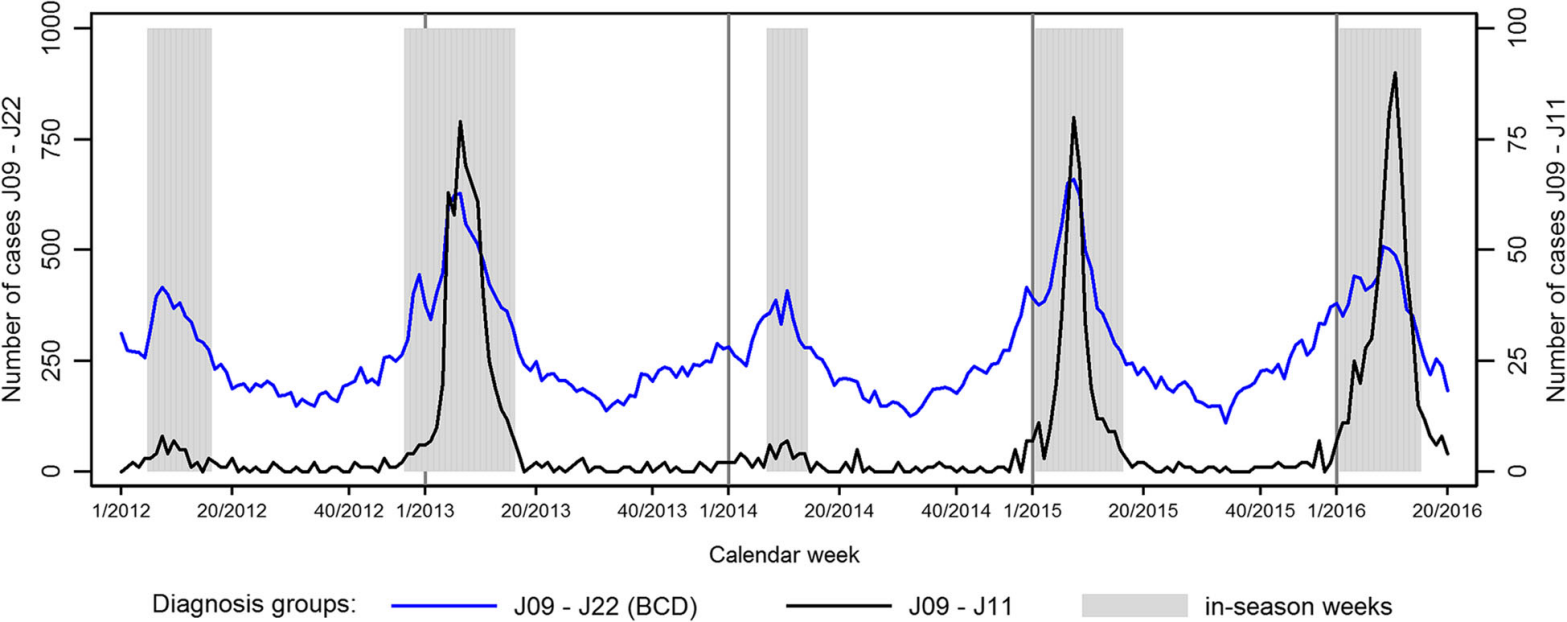
Number of patients with ICD-10 codes J09 - J22 (influenza, pneumonia and other acute lower respiratory infections (BCD), left y-axis) and patients with ICD-10 codes J09 - J11 (influenza, right y-axis) in primary diagnosis per week in 47 sentinel sites from week 1/2012–20/2016, in-season weeks are highlighted in grey color

The explicit in-season primary diagnoses of influenza (J09 - J11) accounted for only 5% of SARI cases fulfilling the basic case definition (Fig. 4). However, during and following peak weeks of influenza seasons 2012/13 (weeks 7/2013–10/2013) and 2014/15 (weeks 8/2015–9/2015), they constituted more than 10%, with a maximum proportion of 18% in the peak of season 2015/16 (week 9/2016). The proportion of influenza-diagnosed patients was also higher in season 2015/16 compared to the previous 4 seasons, and it exceeded 10% for 5 consecutive weeks (weeks 9/2016–13/2016). The proportion of influenza diagnoses among cases meeting the BCD was very low in the seasons 2011/12 and 2013/14 with a maximum rate of 2% influenza diagnoses (J09–11) among all J09–22 primary discharge diagnoses. Off-season influenza diagnoses (J09 - J11) were very rare with a mean proportion of 0.6% in the acute lower respiratory diseases group (J09–22).

### Comparison of case definitions

**Fig. 5:**
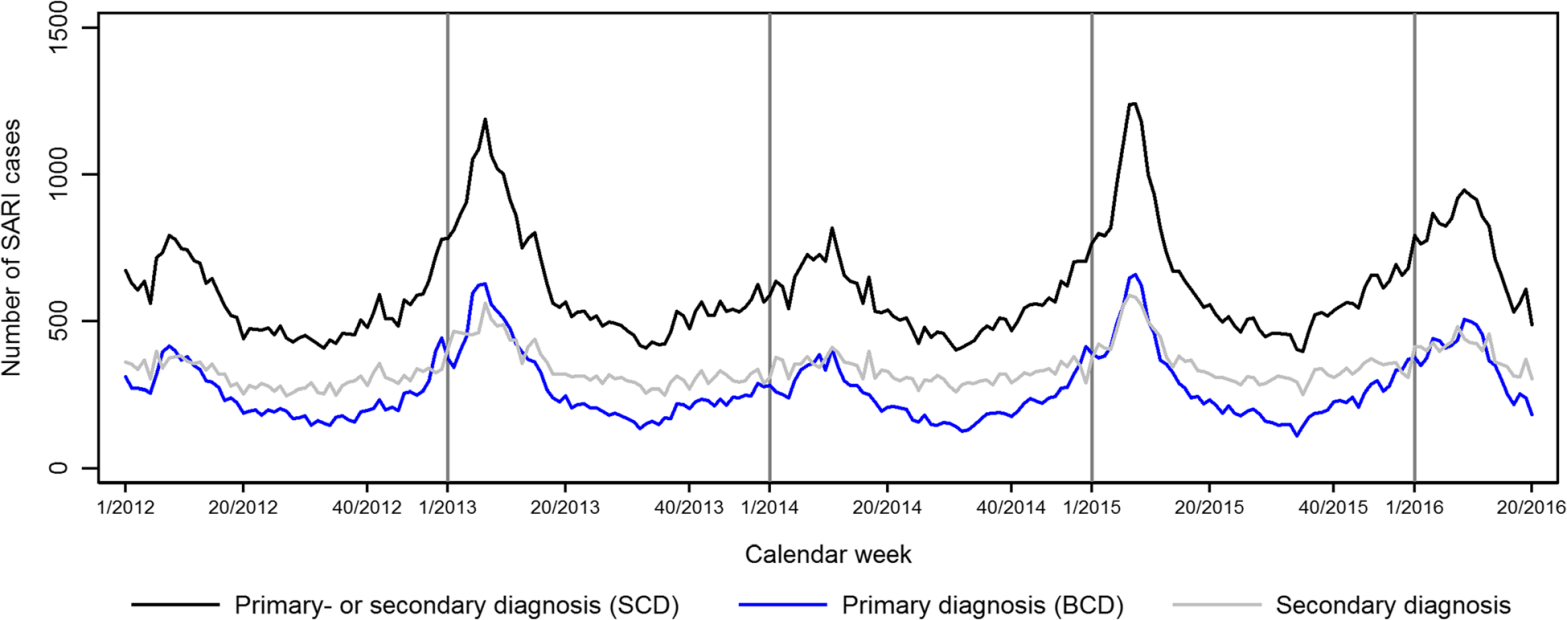
Number of SARI cases with ICD-10 codes J09 - J22 in primary diagnosis (basic case definition, BCD), in secondary diagnosis and in main or secondary diagnosis (sensitive case definition, SCD) per week in 47 sentinel sites from week 1/2012–20/2016

After descriptive exploration of different ICD-10 code groups we selected ICD-10 codes J09–22 for retrospective analysis of SARI cases using the three different case definitions, as defined in the methods section. In order to capture all SARI cases per week in the sentinel hospitals and to estimate the burden of disease, we applied the SCD. The sensitivity of this case definition was higher than for the BCD as it includes not only patients with primary discharge diagnosis from J09 - J22, but also cases with any secondary discharge diagnosis from J09 - J22 (Fig. 5).

Comparison of total numbers of cases identified using each of the case definitions showed that the SCD identified twice as many SARI cases as the BCD and nearly four times as many SARI cases as the TCD (also see Fig. 1). However, seasonality became less obvious when the SCD (any primary or secondary discharge diagnoses from J09 - J22) was applied as compared to the BCD (only primary discharge diagnosis from J09 - J22).

Weekly proportions of hospitalizations, ICU admissions and deaths associated with SARI cases were compared in order to assess the impact and the severity of a season using the different case definitions [28]. The weekly number of all patients hospitalized in the 47 sentinel hospitals fluctuated markedly in the weeks at the end of the year. In weeks outside the Christmas Holidays, a median of 12,934 patients per week were hospitalized (range: 10,327–14,423 patients per week). During Christmas holidays, the median number of admitted patients per week fell to 8054 (range: 6697–9999 patients per week).

**Fig. 6:**
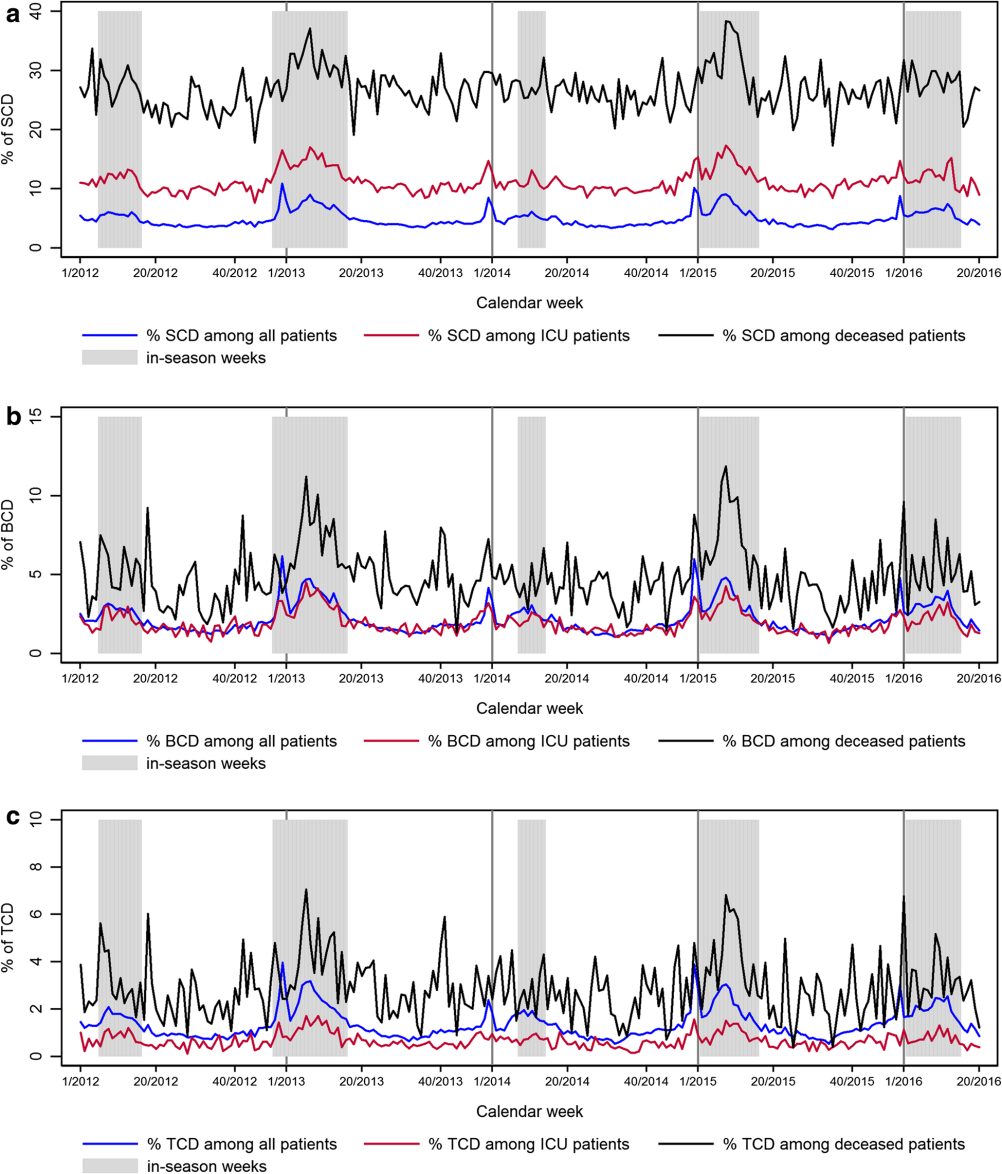
**a**, **b**, **c** Proportion of hospitalized patients, hospitalized patients admitted to ICU and hospitalized deceased patients fulfilling the sensitive case definition (SCD, **a**), basic case definition (BCD, **b**) or timely case definition (TCD, **c**) per week in 47 sentinel sites from week 1/2012–20/2016, in-season weeks are highlighted in grey color

The median proportion of patients per week fulfilling the SCD among all hospitalized Patients was 4% off-season and 6% in-season with peaks of up to 11% during Christmas holidays and during the influenza season (Fig. 6a). Among patients admitted to the intensive care unit (ICU) during their hospital stay, a median of 10% fulfilled the SCD in the off-season rising to 13% during the influenza season. Peaks up to 17% were observed during the influenza season and Christmas holidays. The proportion of SCD patients among deceased was much more variable over the year, since the numbers were much smaller per week. The median proportion of cases per week among deceased was 26% in the off-season and 29% in-season and during Christmas holidays. It ranged from 17% to a maximum of 38% in the 2014/15 influenza epidemic.

The median proportion of patients fulfilling the BCD however was 2% for both all inpatients and those admitted to the ICU (Fig. 6b). Similar to the proportion of SCD, there were peaks during holidays and during the influenza season. In contrast, the median proportion of BCD among deceased patients was higher compared to the overall proportion of cases among all patients. It was 4%, ranging from 1% to 9% in the off-season, and 6% ranging from 3% to 12% during the influenza season with two distinct peaks in the strong influenza seasons of 2012/13 and 2014/15.

The proportions of patients fulfilling the BCD and TCD for the groupings of: all patients, patients admitted to ICU and deceased; followed a similar pattern over time (Fig. 6b and c). However, a smaller proportion of patients fulfilled the TCD compared to the BCD in the groupings: all patients, patients admitted to ICU and deceased patients. The proportion of patients fulfilling the TCD was particularly low for patients in ICU where patient stays are likely to be longer due to greater illness severity thus exceeding the time restriction.

With the implementation of weekly data reporting in the 2015/16 surveillance we were able to examine the data for time lags in reporting. In the data from week 1/2012 to week 20/2016, 99% of the SCD were hospitalized for less than 64 days, which was a little more than 9 weeks. In contrast to that, 99% of the BCD had been released after only 33 days of hospitalization. This meant that reporting on cases hospitalized in a specific week was nearly complete after 10 weeks.

Using the admission week 6/2016 we found that within 2 weeks (week 8/2016) 62% of the BCD and 83% of the TCD had been reported (Table 1). A smaller proportion, 45% of cases fulfilling the SCD, had been reported by this timepoint. As the TCD had restrictions on the hospitalization time (less than 8 days), we had the complete report on cases after 3 weeks. However, data reported only one week (week 7/2016) after week 6/2016 included less than 8% of the cases compared to the reference dataset (week 16/2016) for all case definitions.

**Table 1:** Completeness of data on cases hospitalized in week 6/2016 in the data reports of weeks 7–9/2016

	Number of BCD in week 6/2016 (percentage of total)	Number of SCD in week 6/2016 (percentage of total)	Number of TCD in week 6/2016 (percentage of total)
Data of week 7/2016	35 (5%)	53 (3%)	35 (7%)
Data of week 8/2016	464 (62%)	694 (45%)	419 (83%)
Data of week 9/2016	671 (89%)	1142 (75%)	503 (100%)
Data of week 16/2016	750 (100%)	1530 (100%)	503 (100%)

Data report of week 16/2016 was the reference dataset

**Fig. 7:**
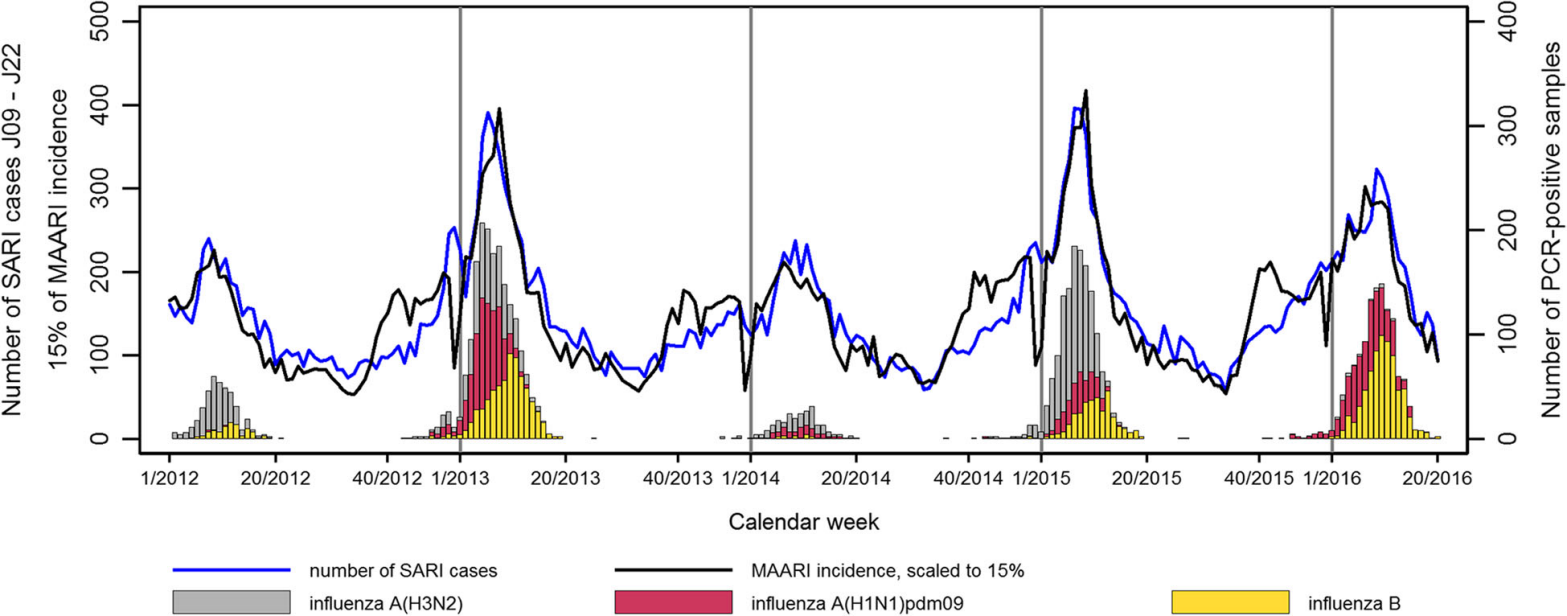
Number of SARI patients with ICD-10 codes J09 - J22 in primary diagnosis and hospitalization 1 week or less (timely case definition, TCD) per week in 47 ICOSARI sentinel sites, MAARI incidence (scaled to 15% of its value) and number of influenza-positive AGI sentinel samples from outpatients, week 1/2012–20/2016

The TCD was compared to the weekly primary care sentinel data on ARI activity within the influenza season in order to evaluate whether it was sufficiently sensitiveto detect changes and peaks in seasonal activity. The peaks in the trend of the TCD matched the time course of the MAARI incidence and the corresponding influenza-positive samples generated by primary care data very well (Fig. 7). The MAARI incidence sloped earlier, around week 40, and had a drop at the end of the year during the Christmas holidays. The inpatient data showed no such rise in autumn, but had a small peak during the Christmas holidays.

### Differences in CD in age, sex, severity level and length of stay

The difference between CD regarding the proportion of SARI cases among all hospitalized patients in different age groups and the proportion of males among SARI cases was presented in Table 2. The proportion of SARI cases was highest in the youngest age group (0–4 years) for all CD. The proportion of SARI cases in the elderly age group (60 years and more) was much higher when applying the SCD.

**Table 2:** Proportion of SARI cases among total hospitalizations and proportions of male cases among SARI cases using different case definitions in 5 age groups and over all age groups in weeks 1/2012–20/2016

	% SARI cases among all hospitalized patients						% male cases among SARI cases					
Case definition	< 5	5–14	15–34	35–59	60+	all	< 5	5–14	15–34	35–59	60+	all
BCD	8.3	2.9	0.8	1.1	2.2	2.2	58.7	53.0	52.9	56.1	53.5	55.1
SCD	9.5	3.6	1.5	2.7	6.3	4.9	58.9	53.8	52.1	60.5	55.1	56.2
TCD	7.2	2.5	0.7	0.7	1.1	1.4	58.6	53.8	51.8	54.5	54.0	55.5

Age-grouped proportions of SARI cases that received ICU treatment, ventilation or had a fatal outcome were presented in Table 3 for the three case definitions. SARI cases fulfilling the SCD had a much higher proportion of severe and fatal outcome. Especially in SARI cases of the two highest age groups, the severe outcome was more pronounced compared to the younger age groups irrespective of the CD used. The proportion of fatal outcomes was especially high in the age group of 60 years and older across all groups. The median duration of stay varied with age and level of care (ICU, ventilation) (Table 4).

**Table 3:** Proportions of ICU treatment, ventilation and deceased among SARI cases using different case definitions in 5 age groups and over all age groups in weeks 1/2012–20/2016

	% SARI cases with ICU treatment						% ventilated among SARI cases						% deceased among SARI cases					
Case definition	< 5	5–14	15–34	35–59	60+	all	< 5	5–14	15–34	35–59	60+	all	< 5	5–14	15–34	35–59	60+	all
BCD	4.6	5.9	7.5	13.9	17.1	12.7	1.3	3.0	2.9	5.8	6.9	5.0	0.02	0.07	0.3	1.5	8.0	4.5
SCD	6.0	8.2	19.6	36.1	38.4	32.6	2.1	3.8	9.3	19.3	17.4	15.1	0.08	0.14	1.7	7.6	15.1	11.3
TCD	2.7	2.9	5.0	8.2	11.1	7.0	0.5	1.2	1.2	2.5	3.7	2.1	0.01	0.04	0.2	1.6	10.2	4.4

**Table 4:** Median duration of hospitalization, ICU treatment and ventilation among affected SARI cases using different case definitions in 5 age groups and over all age groups in weeks 1/2012–20/2016

	Median duration of stay (days) for SARI cases						Median duration in ICU (days) for SARI cases with ICU treatment						Median duration of ventilation (hours) for ventilated SARI cases					
Case definition	< 5	5–14	15–34	35–59	60+	all	< 5	5–14	15–34	35–59	60+	all	< 5	5–14	15–34	35–59	60+	all
BCD	4	4	4	6	8	6	3	4	3	3	3	3	100	140.5	78	74	61	69
SCD	4	4	5	8	10	8	3	4	4	5	4	4	118	143	128.5	142	95	104
TCD	4	4	3	4	5	4	2	3	1	1	1	2	44	117	47	41.5	32	37

## Discussion

Our analyses of data on SARI cases demonstrated the potential of the newly established syndromic hospital surveillance system. For the first time it was possible to show the number and proportion of SARI cases in hospital as a time series over five subsequent influenza seasons in Germany. The temporal pattern of hospitalized SARI patients in sentinel data corresponded well to the course of the MAARI incidence and the virological results of the German outpatient sentinel (AGI). The earlier rise of the MAARI incidence in autumn compared to SARI case frequency can be attributed to Rhinovirus circulation in this time of the year, as had been shown in the seasonal influenza reports from the primary care sentinel surveillance (AGI [5]).

As the aim of this study was to establish a robust sustainable SARI surveillance system, we not only tested different diagnosis groups in order to determine an optimal combination of ICD-10 codes from chapter J, but also different diagnosis classes (admission or discharge; primary or secondary) and time restrictions in the length of stay. We found that no single case definition is able to meet all our surveillance requirements optimally, as had been discussed before [29].

We were able to show that a very specific selection of ICD-10 codes including only patients with primary diagnosis J09 to J11 is not suitable for surveillance purposes by itself. Mild influenza seasons such as 2013/14 would not have been detected using this definition. This disadvantage cannot be adjusted by an additional inclusion of patients with secondary diagnosis J09 to J11 (data not shown). As there are no specific recommendations for influenza diagnostic in Germany, it is up to the attending physician to decide if a patient with acute respiratory symptoms will be tested for influenza. By using only ICD-10 codes J09–11 the overwhelming proportion of influenza and influenza-related illness of the lower respiratory tract will be missed due to testing bias. Therefore, we used an ICD-10 grouping that captures both the influenza-related pneumonias and other influenza-related acute infections of the lower respiratory tract as recommended for syndromic influenza surveillance [14, 28].

The basic case definition (BCD) using only primary discharge diagnoses of acute lower respiratory infections (J09 – J22) is the only CD that can be used for comparisons with other health statistics of hospitalized patients both nationally and internationally. But using only primary diagnoses captures mainly the population without major comorbidities. Therefore, a more sensitive case definition (SCD) was found to be more appropriate in terms of estimating disease burden. SARI patients with an underlying chronic disease such as heart disease or chronic respiratory disease may have their comorbidity coded in the primary diagnosis and the acute respiratory disease in their secondary diagnosis, as the aggravation of the chronic disease could play a major role in their hospitalization [30]. This means that an after-season assessment of severity and burden could be used to compare BCD and SCD in order to provide additional information on the populations most affected.

The SCD is not suitable for in-season surveillance of SARI activity, as patients with underlying diseases tend to have a more extended stays in hospital and their data is available later than data of patients with no or only minor illnesses apart from their acute respiratory disease. The subset of SARI patients fulfilling the BCD with a length of stay <8 days i.e. meeting the timely case definition (TCD) were used to assess the trend of acute respiratory activity. The TCD data lags two weeks behind the information from primary care sentinal surveillance (AGI), but still allows a timely situation assessment within the season.

Ideally this information would be available even earlier by using the admission diagnosis while patients are still hospitalized. It was not possible to establish this at the beginning of the project. However, about two-thirds of SARI patients fulfilling the BCD at discharge also had an admission diagnosis code from J09 - J22 . This proportion remained relatively stable throughout the study period (data not shown). Using the admission diagnosis with the diagnosis codes we selected (J09 - J22) would enable a nearly real-time hospital surveillance in the future [14, 28].

The available data set allowed an exploratory approach and provided additional information, also on potential country-specific issues like changing hospitalization rates over the Christmas period. Many primary care practices are closed during the holidays and patients with acute illness tend to go to hospital directly while planned surgeries and examinations in hospitals are postponed. Our findings of consistently higher hospitalization rates over the Christmas holidays might be valuable in the interpretation of the data in future epidemics as they might lead to overestimation of the burden of acute respiratory disease at this time of the year.

We included additional information, such as the duration of stay in normal wards, ICU and the duration of ventilation by age group. This has been shown to provide valuable information on the impact of the secondary health care level during severe influenza seasons [31, 32]. The proportion of SARI cases with fatal outcome in the severe influenza A (H3N2)-dominated 2014/15 season corresponded well with the results of the estimated excess mortality in other European countries [33].

Only a very small fraction of hospitalized patients with acute respiratory symptoms are tested for laboratory confirmation of influenza in Germany [9]. This may have different reasons. Firstly, onset of disease starts several days before the symptoms get worse and family doctors refer patients to hospital. At this point secondary bacterial infection may have become the focus of diagnostic and treatment and influenza viruses can no longer be identified [34, 35]. Secondly, clinical influenza diagnosis as selection criterion for laboratory confirmation may only detect patients that display typical influenza-like-illness. Fever as a principal symptom loses its significance especially in elderly patients [36–38]. Thirdly, 75% of community acquired pneumonia is diagnosed and ICD-10-coded without identification of a causative pathogen in Germany (J18.-) [39].

For these reasons we decided to adopt the surveillance recommendations of the WHO [14] for a sensitive SARI case definition. A comparable sensitive ICD-10-based ARI case definition was implemented successfully in the primary care syndromic surveillance [5, 20]. However, specific and representative information on influenza virus circulation is absolutely necessary for the interpretation of the data according to the impact of influenza on SARI cases. Therefore the results of the national virological sentinel surveillance of the primary care sentinel (AGI) are relayed on a daily basis from the National Influenza Reference Center (NIRC) to the epidemiological influenza database of our unit at the RKI [5].

One of the most important limitations of our approach was the current lack of complementary virological information, with testing of at least a representative subset of the identified SARI patients, as recommended by the WHO [14]. Where an investigation specifically focusses on laboratory confirmed influenza, we could restrict our analysis to patients with ICD-10 diagnoses J09 and J10 (Influenza due to identified influenza virus). However, a complementary virological inpatient surveillance system could be established in the future.

## Conclusions

We were able to show that the available data and reporting procedures implemented, provided timely and reliable information on SARI in inpatients in Germany. The exploratory approach gave valuable insights into the data structure, showed the importance of using different case definitions tailored to the objectives of the evaluation, and allowed the adaptation of our country specific data sources according to international recommendations. Based on the five seasons analyzed we were able to create a SARI baseline as a starting point for evaluation of severity of future influenza epidemics or pandemics. At the same time, the newly established system will allow an even more detailed analysis focusing on different subgroups of patients with specific risk factors and underlying conditions.

## Appendix

**Table 5:** Selection of potential SARI case definitions after literature review

ICD-10 Coding [18]	Description of potential SARI case definitions
J09.x-J10.x	Influenza, laboratory confirmed
J09.x-J11.x	Influenza
J12.x-J18.x	Pneumonia
J09.x-J18.x	Influenza and pneumonia
J09.x-J22.x	Influenza, pneumonia, acute lower respiratory tract infection [26, 27]
J00.x-J22.x	Acute respiratory disease
J11.x, J18.9, J20.9, J20.88, J09.x, B34.5, R50.9	ILI, including unspecific viral Infektion and unspecific fever [25]
J00.x-J03.x, J40.x-J44.x, J47.x	ARI, general [24]
A15.x, A16.x, A20.2, A21.2, A22.1, A37.x, A38.x, B34.x, J00.x-J32.x, J35.x-J37.x, J39.x-J91.x, J93.x-J94.x, J96.x-J98.x, R04.x-R07.x	Respiratory disease, including bioterrorism [27]

## Abbreviations

AGI: Arbeitsgemeinschaft Influenza/Working Group Influenza
ARI: Acute respiratory infections
BCD: Basic case definition
CD: Case definition
ICD-10: International Statistical Classification of Diseases and Related Health Problems 10th Revision
ICU: Intensive care unit
MAARI: Medically attended acute respiratory infections in outpatients
PHEIC: Public Health Emergency of international Concern
PIKS: Pandemische Influenza Krankenhaus-Surveillance/Pandemic Hospital Based Surveillance
RKI: Robert Koch Institute
SARI: Severe acute respiratory infections
SCD: Sensitive case definition
TCD: Timely case definition
